# When Do Korsakoff Patients Justify Immoral Behaviors? The Influence of Premorbid Delinquency and Self-Other Perspectives in Moral Decision-Making and Moral Reasoning

**DOI:** 10.3390/jcm12196257

**Published:** 2023-09-28

**Authors:** Nairobi Vlot, Albert Postma, Erik Oudman

**Affiliations:** 1 Experimental Psychology, Helmholtz Institute, Utrecht University, 3584 CS Utrecht, The Netherlandsa.postma@uu.nl (A.P.); 2 Lelie Care Group, Slingedael Korsakoff Expertise Center, Slinge 901, 3086 EZ Rotterdam, The Netherlands

**Keywords:** Korsakoff’s syndrome, moral decision-making, moral reasoning, delinquency

## Abstract

Korsakoff’s syndrome (KS) is a chronic neuropsychiatric disorder caused by a vitamin B1 deficiency. KS is characterized by profound amnesia and often accompanied by poor executive functioning, decreased social-cognitive abilities, and difficulties in behavioral regulation. As moral behaviors and attitudes may provide insight in socio-behavioral interactions, the current study aimed to evaluate everyday moral maturity by administering self- versus other-oriented moral dilemmas in a group of KS patients (n = 20) and healthy controls (n = 20). Responses were scored according to the Kohlberg stages of moral reasoning. Furthermore, we assessed premorbid delinquency and current neurocognitive functioning as possible relevant factors. Our results show that KS patients were prone to lower levels of moral maturity when confronted with moral dilemmas relating to themselves, compared to dilemmas relating to (un)personal others in KS patients, while healthy subjects showed an opposite pattern. Moral immaturity could find its origin already before the onset of the KS diagnosis, as suggested by the elevated premorbid levels of delinquent behavior and correlation between premorbid delinquency and moral maturity in KS. Lower moral maturity could therefore be a possible predisposing factor to both delinquency and later development of Korsakoff’s syndrome.

## 1. Introduction

Korsakoff’s syndrome (KS) is a chronic neuropsychiatric disorder caused by a thiamine (vitamin B1) deficiency, most frequently caused by alcohol dependence and malnutrition [1]. KS is associated with cognitive disorders, such as severe declarative memory disorder and impairments in executive functioning [2]. Moreover, neuropsychiatric symptoms are prevalent, such as irritability, aggression, and disinhibition of behavior [3,4]. There is increasing evidence that KS patients also show social and personality changes [3,5]. Alterations in social behavior may include a higher prevalence of apathy [6], social isolation and loneliness [7], along with difficulties regulating own behavior and maladaptive interpersonal interactions [5,7,8]. Possible explanations for social dysfunctions in KS can be found in a deterioration of primary social skills, such as emotion recognition [8,9]. Subsequently, this may lead to inadequacy in more elaborate socio-cognitive skills, such as empathy, perspective-taking, theory of mind, and understanding faux-pas situations [8,10]

A crucial ability to behave and decide in a socially adequate manner comprises moral decision-making. It refers to ‘making judgements within the moral domain’ [11]: making decisions on dilemmas regarding moral issues or principles such as justice, harm, fairness, and care [12]. According to the dual-process model of moral decision-making [13], two forms of moral decision-making can be differentiated: deontological versus utilitarian decision-making [14]. Deontological decision-making is based on the idea that the morality of an action depends on the intrinsic nature of the action (e.g., harming others is wrong regardless of its consequences). Responses to moral dilemmas are based on (cultural) norms and therefore judgement is considered often rapid and automatic. In contrast, utilitarian moral decision-making requires more reasoned processing and such decisions are founded on the idea that the morality of an action is determined by its consequences (e.g., harming others is acceptable if it increases the well-being of a greater number of people). Thus, obeying (moral) rules is taken less strictly when judging in a utilitarian way [14].

Research has pointed to different biases for either form of decision-making in various clinical populations. For instance, patients with selective bilateral hippocampal damage show a tendency towards deontological responses, while patients with traumatic brain injuries show a bias towards utilitarian responses [15,16]. Moreover, certain personality traits appear to be associated with utilitarian responses, namely antisocial personality- and psychopathic traits [17]. Individuals with alcohol use disorder (AUD) also seem to show a tendency towards utilitarian response in personal moral dilemmas when compared to controls [18,19]. In KS, a utilitarian bias was not found in everyday and abstract moral dilemmas, which suggests relatively preserved moral decision-making abilities in this population. Remarkably, their moral reasoning abilities were not fully intact [20]. The cause of this discrepancy between moral decision-making and moral reasoning is yet to be explored, although possible explanations could be low pre-morbid capacities, as well as the extent to which dilemmas have negative (or positive) consequences for the individual itself [20]. This latter explanation would suggest that judging own versus others’ behavior comprises different processes, thereby possibly leading to different moral outcomes. A study [21] supports a discrepancy between self- versus other-oriented moral decision-making on a neural level, thereby implying that moral decisions might be influenced by personal outcomes.

Recent investigations into moral decision-making in patient groups indicated that not only the decision itself is of relevance, but also the motivation behind this decision—which can be described as moral reasoning [22]. According to the developmental model of Kohlberg [23], moral reasoning abilities develop in different stages throughout the lifespan. The first stage is referred to as ‘preconventional reasoning’, where judgement is solely based on the needs and perceptions of the actor (the individual itself). Preconventional arguments are characterized by egocentrism, focus on the self-perspective, and the avoidance of punishments. The second stage of reasoning is named ‘conventional’, in which considerations of society and society’s laws are taken into account. Here, obeying rules and being good and nice to others play an important role. The final stage is called ‘postconventional’, in which judgements are based on abstract and ingrained personal principles that guide decision-making. Judgements are not necessarily defined by laws but are deliberate and based on own core beliefs. According to Kohlberg, only 10–15% of individuals will reach the postconventional reasoning stage [23].

Lower moral maturity, reflected as lower moral reasoning skills, could be a consequence of brain alterations, for example, resulting from poor social cognitive skills, as moral behavior requires involves social-cognitive processes [22,24]. Moreover, specific aspects of executive functioning are considered to have predictive value for low moral reasoning abilities, among which cognitive flexibility [25]. Alternatively, lower moral reasoning skills might also be a predisposing factor to brain alterations. One suggestion for this idea could be elevated levels of criminal behaviors in their past. To illustrate, a failure to follow moral guidelines is characteristic of antisocial, rule-breaking behavior [26], leading to a bias in utilitarian decision-making in this population [17]. Regarding moral reasoning, a recent meta-analysis provided strong evidence for a link between juvenile delinquency and lower moral reasoning abilities [27], suggesting the possibility that delinquency could be a token of diminished moral behaviors. To our current knowledge, this link has unfortunately only been studied in adolescents, but not in populations with neurological or (neuro)psychiatric deficits. Along with a presumably high prevalence of AUD and KS in the criminal justice system [28,29], the result raises the question if lower reasoning abilities and a higher level of (trait) delinquency also hold for adult KS patients with a criminal history.

Considering the foregoing, the current study aimed to evaluate possible differences in moral decision-making and moral reasoning between KS patients and age-, gender-, and education-matched healthy controls by administering everyday moral dilemmas. Adding to previous research [20], this study provided dilemmas with three different perspectives: self, an unpersonal other, and a personal other, to explore the influence of avoiding negative consequences for the individual self, as compared to negative consequences for others. Moreover, this study aimed to explore whether current moral abilities can be related to predisposing factors (i.e., premorbid delinquency) or current neurocognitive functioning. Emotion recognition abilities, which are considered a primary social cognitive skill, and cognitive flexibility were assessed in this regard. We expected KS patients to judge moral situations as ‘less wrong’ as compared to healthy controls when dilemmas were beneficial to themselves (in contrast to others). Furthermore, we hypothesized that KS patients were more intent on performing the actual dilemmas when compared to healthy controls (HC), thereby reflecting a utilitarian bias that was also present in other patient groups. Regarding moral reasoning, we hypothesized that KS patients would show lower moral reasoning abilities than the healthy controls in all three conditions, but particularly when situations would be beneficial to themselves. We expected a correlation between premorbid delinquency and lower moral reasoning abilities.

## 2. Materials and Methods

### 2.1. Participants

A total of 20 patients (14 male) diagnosed with KS participated in this study. Demographic variables are described in Table 1. All KS patients were inpatients of a long-term care facility and fulfilled the DSM-V criteria for alcohol-induced major neurocognitive disorder, Amnestic Confabulatory type (code: 291.1) [30]. Extensive neuropsychological research was performed to establish the KS diagnosis prior to this project. At the time of testing, patients were in the chronic stage of KS and not in the Wernicke Encephalopathy. Patients were abstinent from alcohol for at least six months. Exclusion criteria were additional neurological disorders (such as TBI, epilepsy, stroke, brain tumor, and dementia), acute psychiatric conditions interfering with the testing procedure, and an estimated IQ below 75. Twenty age-, education-, and gender-matched healthy participants (14 male) were included as a reference group (See Table 1). Recruitment took place via various public platforms (e.g., Facebook, LinkedIn, and social network). Similar exclusion criteria applied: the presence of neurological disorders, acute psychiatric conditions, and an estimated IQ below 75.

The study was conducted according to the Declaration of Helsinki and ethical approval was obtained. A written informed consent form was obtained by all participants and, for KS patients, also by their legal representatives.

### 2.2. Materials

#### 2.2.1. Moral Behavior Inventory

The Moral Behavior Inventory (MBI) was used to assess moral decision-making and moral reasoning abilities. It consists of 24 short dilemmas, reflective of daily life [31]. This questionnaire was used to assess participants’ knowledge of everyday social and moral norms in addition to moral reasoning skills. Examples of items are “cut in line when in a hurry” and “take the last seat on a crowded bus”. As in earlier research, items 4, 9, 20, and 21 were excluded based on inappropriateness for the KS patient group. Item 4 (‘drive after having one drink’) refers to a skill (i.e., driving a car) that not every KS patient has attained, but also to specific disease-related behavior prior to KS. This item was rephrased to a delinquent behavior in the Delinquent Behavior Questionnaire. Item 9 (driving out homeless people from the community) can be considered offensive to KS patients, as some may have experienced homelessness. Items 20 and 21 refer to situations that do not commonly occur in the lives of KS patients (doing homework, having jury duty) [20].

The traditional version of the MBI was designed for a personal view, i.e., ‘self’-centered. For the current study, dilemmas were rewritten by the researchers to add two other viewpoints: a personal other and unpersonal. For the ‘unpersonal other’ condition, a fictional but credible person was introduced. Participants were informed of the name, age, and hometown of this person and the notion that he/she was in good health. Gender of the fictitious person was matched with the gender of the participant. After this, it was checked whether the fictive person was indeed not acquainted to the participant. In the ‘personal other’ condition, participants were asked to name a friend or friendly acquaintance. This led to rephrased dilemmas, for example: “how wrong would you rate [name of person] to park on a disabled parking space?”.

The researcher read the dilemmas aloud once before inviting participants to make a judgement about the degree of wrongness (moral decision-making) on a four-point Likert scale (1—not wrong, 2—mildly wrong, 3—wrong, and 4—completely wrong). Subsequently, participants were asked to provide a reasoned justification for their choice, reflecting moral reasoning. The answers were written down and scored according to the corresponding reasoning level, consistent with the moral development theory of Kohlberg [23] (1—preconventional, 2—conventional, and 3—postconventional). Reasoning level was determined by calculating the average score of all 20 items. In the ‘personal other’ and ‘unpersonal other’ conditions, the ability to understand items was scored binomially per item (0—no understanding or 1—understanding) and subsequently added up into a sum score per condition. In the ‘self’-condition, participants were asked if they would perform the dilemma themselves.

#### 2.2.2. Emotion Recognition Task

Emotion recognition abilities were assessed by means of a facial emotion recognition task: the Emotion Recognition Task (ERT) [10]. This is a computerized test consisting of emotionally salient morphs (i.e., short video fragments) of faces, gradually increasing in intensity. Faces are portrayed by Caucasian males and females of whom characteristics, such as hair and clothing, are not visible. Intensity increases in five levels: 0%, 40%, 60%, 80% and 100%, similar to how emotions typically develop. In each item, participants must select which of the six basic emotions is portrayed (fear, sadness, happiness, disgust, anger, and surprise) (Lysaker et al., 2020). The ERT is validated in neurological and psychiatric populations, among which are KS patients [9]. After completing the test, a total score was obtained for each participant based on education, age, and gender. This score was subsequently compared to norm data by the computer, leading to a calculated residual score. Residual scores were used as an interacting variable in the current study.

#### 2.2.3. D-KEFS Color Word Interference Test and BADS Rule Shifts Card Test

The D-KEFS Color Word Interference Test (CWIT; [32]) and the Rule Shifts Card Test, a subtest of the Behavioral Assessment of the Dysexecutive Syndrome (BADS [33]), were used to assess cognitive flexibility. In the current study, scaled scores based on completion time and error scores of the fourth condition of the D-KEFS CWIT were computed and used as an interacting variable. Reliability and validity of the CWIT test are sufficient to high [32,34].

#### 2.2.4. Delinquency Questionnaire

For the current study, the “Delinquency Questionnaire” was developed as an adaptation of the ‘International Self-Report Delinquency Instrument’ (ISRD; [35]) and the ‘Self Report Delinquency Scale’ (SRDS; [36]), which were originally designed for the youth and adolescent populations. The “Delinquency Questionnaire”, a self-report measure, contains 12 statements regarding engagement in delinquent behavior in one’s personal life. Six items refer to violent offences, e.g., ‘Throughout my life, I have injured someone with a weapon’. The other six items relate to non-violent offences, e.g., ‘Throughout my life, I have traded in, or produced drugs’. Items were scored based on frequency of eventually performed behavior—never (0), once (1), or more than once (2). The total score was used as a measure of delinquent behavior.

### 2.3. Procedure

**Figure 1:**
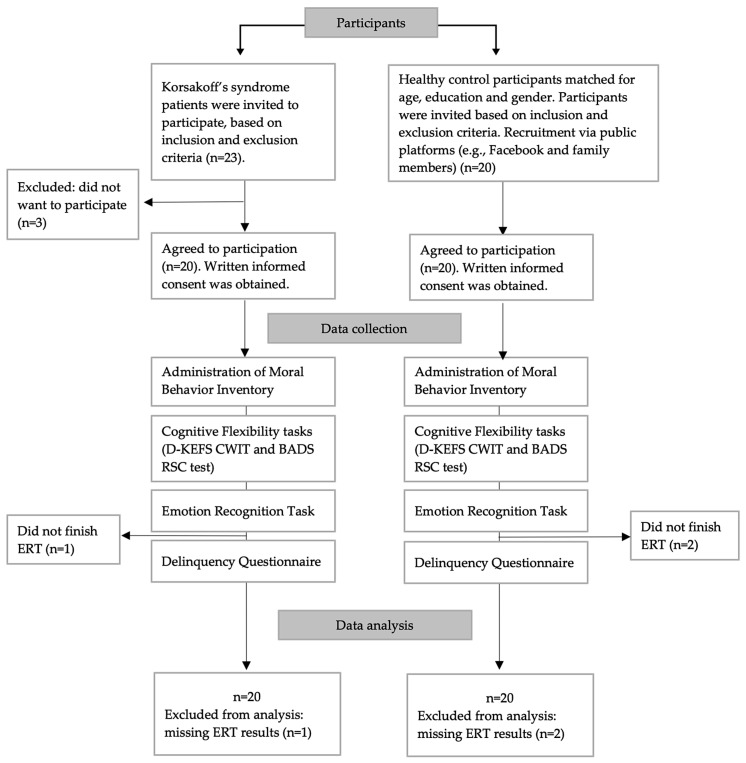
Flowchart of study design. Notes: D-KEFS CWIT = D-KEFS Color Word Interference. Task; BADS RSC Test = BADS Rule Shifts Card Test; ERT = Emotion Recognition Task.

See Figure 1 for an overview of the procedure. Total duration of administration was 60 to 75 min. The testing session was debriefed.

### 2.4. Data Analysis

The current study is a mixed design.

Independent sample *t*-tests were used to compare the two groups (KS and HC) based on age, total emotion recognition, justifications of self-centered dilemmas, and moral decision-making in the three conditions of the MBI. The assumption of normality was met in these analyses. Due to the violation of assumptions of normality and the assumption of heterogeneity of variances, nonparametric Mann–Whitney U tests were used to evaluate differences in the two tests’ measuring cognitive flexibility (BADS Rule Shift Cards Test and D-KEFS Color Word Interference Test, condition 4), and the Delinquency Questionnaire.

Understanding was binomially scored as 0 (no understanding) or 1 (understanding) and was considered a prerequisite for thoughtful reasoning on moral dilemmas. The absence of understanding was per definition noted as a low (level 1) reasoning level. However, a level 1 reasoning is not equal to a lack of understanding. Therefore, for comparison purposes, reasoning levels corrected for understanding will be used in the data analysis, i.e., the average reasoning level was calculated over the number of items that were understood.

A 3 × 2 mixed model ANOVA was used to investigate the stability in moral reasoning level in three conditions of the MBI between KS patients and HC. Mauchly’s test indicated that the assumption of sphericity has been met, *χ*^2^(2) = 4.587, *p* = 0.101.

Due to violations of the assumption of normality, Spearman’s rho correlations were calculated for emotion recognition and understanding, emotion recognition and delinquency, delinquency and moral decision-making, cognitive flexibility and moral decision-making, and moral reasoning and delinquency. Correlations were calculated for each group. Scaled error scores of the D-KEFS Color Word Interference Test (4th condition) were eventually used as a measure for cognitive flexibility, as mean group difference on this performance was largest, thereby having the highest sensitivity. The possibility of a linear relationship between delinquent behavior and reasoning levels in three conditions was assessed by calculating bivariate Spearman correlations.

**Table 1:** Participant characteristics and scores on neuropsychological tasks per group.

Characteristics	KS (n = 20)	HC (n = 20)	Statistics
Gender (m, f)	14 (6)	14 (6)	χ^2^(1, N = 40) = 0.00, *p* = 1.00
Age (years; M, SD)	61.70 (6.47)	61.55 (6.34)	*t*(38) = 0.074, *p* = 0.941
Age (years; Minimum, Maximum)	47 (74)	47 (73)	
Educational Level (M, SD)	4.45 (1.47)	4.65 (0.67)	*U =* 181.50, *z* = −0.53, *p =* 0.594
Rey AVL Test—inprenting percentile score (M, SD)	4.3 (5.94)		
Rey AVL Test—recall percentile score (M, SD)	2.05 (4.31)		
VAT percentile score (M, SD)	10.4 (17.24)		

Notes: m = male; f = female; n = number; HC = Healthy Controls; KS = Korsakoff Patients; M = mean; SD = standard deviation. Educational level was assessed in seven categories: 1 (primary school); 7 (academic degree) [37]; Rey AVL = Rey Auditory Verbal Learning Test [38]; VAT = Visual Association Test [39].

## 3. Results

### 3.1. Demographic Variables

Descriptive characteristics for the KS and HC groups are summarized in Table 1. Groups did not significantly differ in gender, age, and educational level.

### 3.2. Basic (Interacting) Variables

Group differences in interacting variables are presented in Table 2. KS patients had lower emotion recognition performances than healthy controls, as can be viewed by ERT performance, and cognitive flexibility—as illustrated by the BADS and D-KEFS scores. Moreover, KS patients reported more violent and non-violent delinquent behavior than the healthy control subjects.

#### Emotion Recognition and Delinquency

Results indicate the absence of a correlation between emotion recognition abilities and delinquent behavior in the KS group, r_s_ = −0.17, *p* = 0.500, two-tailed, N = 19, and the HC group, r_s_= 0.312, *p* = 0.207, two-tailed, N = 18. This implies no linear relationship between emotion recognition abilities and delinquency.

### 3.3. Moral Decision-Making

#### 3.3.1. Moral Decision-Making in Self, Personal Other and Unpersonal Other Condition

Healthy controls significantly reported fewer justifications (M = 2.10; SD = 2.05) for dilemmas (i.e., ‘would you imagine doing this yourself?’) than KS patients (M = 5.15; SD = 3.42), t(31.07) = 3.42, *p* = 0.002, two-tailed, d = 1.08, 95% -CI [1.23, −4.87]. In other words, KS patients more often reported ‘yes, I would do this’ on self-directed dilemmas.

Groups significantly differed in moral decision-making in the ‘self’ condition, with the HC group (M = 59.15; SD = 6.05) reporting higher wrongness, 95% -CI [−12.01, −1.39], than KS patients (M = 52.45; SD = 9.95) t(31.38) = −2.57, *p* = 0.015, two-tailed, d = −0.81. This indicates a large effect and implies that HCs judge the execution of moral dilemmas as more wrong than KS patients do.

Groups significantly differed in moral decision-making in the ‘personal other’ condition, with the HC group (M = 58.80; SD = 5.53) reporting higher wrongness, 95% -CI [−17.47, −4.63], than KS patients (M = 47.75; SD = 12.82), t(25.83) = −3.54, *p* = 0.002, two-tailed, d = −1.12. This indicates a large effect and points to higher wrongness in HCs when evaluating the behaviors of a personal other.

**Figure 2:**
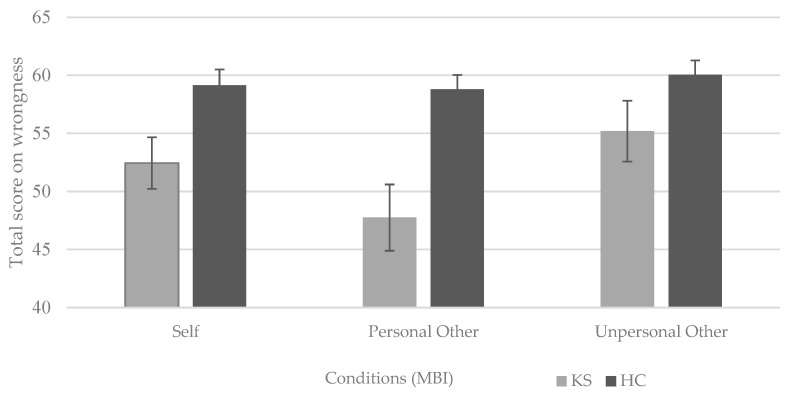
Total scores on judgements of wrongness in Korsakoff’s syndrome patients (n = 20) and healthy controls (n = 20). Bars represent the sum of wrongness (on a 1–4 Likert scale) over 20 items of the Moral Behavior Inventory in the self-perspective (**left**), personal other condition (**middle**), and unpersonal other condition (**right**). Error bars represent standard errors of the mean. Notes: KS = Korsakoff’s syndrome; HC = healthy controls; MBI = Moral Behavior Inventory.

Moral decision-making in the ‘unpersonal other’ condition was not statistically significant, although the HC group (M = 60.05; SD = 5.51) did report a trend towards a higher wrongness, 95% -CI [−10.79, −1.09], than KS patients (M = 55.20; SD = 11.72), t(27.01) = −1.674, *p* = 0.106, two-tailed, d = −0.53. This indicates a medium to large effect (See Figure 2).

#### 3.3.2. Moral Decision-Making, Emotion Recognition, Cognitive Flexibility, and Delinquency

Additional correlations on imaginary performance (i.e., would you perform this behavior?) and wrongness scores of the three conditions of the MBI were performed to further investigate moral decision-making. Correlations with emotion recognition, cognitive flexibility, and delinquency are presented in Table 3. The correlations imply that moral decision-making abilities are unrelated to emotion recognition performance and cognitive flexibility in both KS patients and healthy controls, as well as in all three conditions. Regarding delinquency, a strong and positive correlation was found with the imaginary performance of moral dilemmas in KS patients. This suggests that KS patients who reported higher delinquency scores are more inclined to perform the moral dilemmas. Furthermore, a moderate and negative correlation was found with wrongness scores in the ‘self’ condition in KS patients, pointing to lower wrongness in delinquent KS patients. The wrongness scores of HCs in all three conditions did not correlate with delinquency. The results imply that delinquency is moderate to strongly related to personal (own) moral decision-making in KS patients.

### 3.4. Moral Reasoning

#### 3.4.1. Moral Reasoning in Self, Personal Other and Unpersonal Other Condition

**Figure 3:**
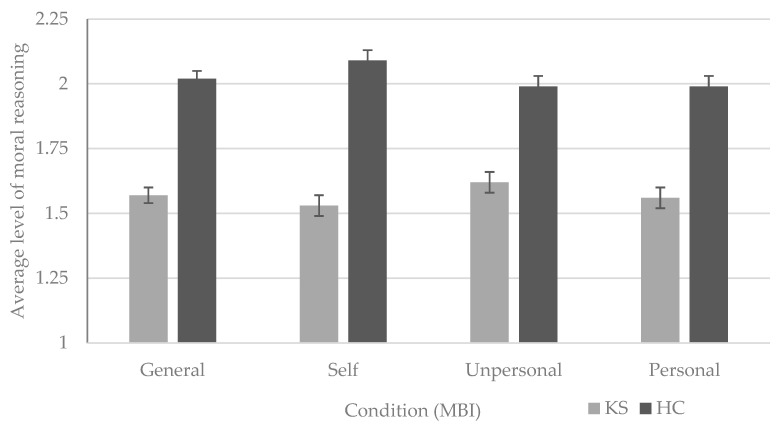
Average level of moral reasoning in Korsakoff’s syndrome patients and healthy controls, with one representing the pre-conventional level, two the conventional, and three the postconventional level. Bars represent the average level of moral reasoning overall (**left**), the self-perspective, unpersonal other condition, and personal condition (**right**) based on 20 everyday moral dilemmas. Error bars represent standard errors of the mean. Notes: KS = Korsakoff’s syndrome; HC = healthy controls; MBI = Moral Behavior Inventory.

No significant main effect for reasoning was found, F (2, 76) = 1.00, *p* = 0.373, partial *η*^2^ = 0.03, suggesting no differences between reasoning levels 1 (preconventional), 2 (conventional), and 3 (postconventional). A significant main effect for group was found, F (1, 38) = 6.14, *p* < 0.001, partial *η*^2^ = 0.72, indicating lower levels of moral reasoning in KS patients compared to healthy controls. A significant interaction between reasoning and group was found, F (2, 76) = 6.08, *p* = 0.01, partial *η*^2^ = 0.14. This shows that KS patients tend to use a lower moral reasoning level when confronted with dilemmas relating to themselves, compared to dilemmas relating to (un)personal others, while HC group shows the opposite pattern (i.e., higher reasoning level for self-dilemmas and lower levels for dilemmas relating to others) (see Figure 3).

#### 3.4.2. Moral Reasoning, Emotion Recognition, Cognitive Flexibility, and Delinquency

Additional correlations on average moral reasoning level in KS patients and HCs were calculated (See Table 4). Regarding emotion recognition, only in the ‘unpersonal other’ condition, a strong and negative correlation was found in the HC group. Cognitive flexibility performance was also only related to moral reasoning in the ‘unpersonal other’ condition. Here, lower moral reasoning in KS patients was related to lower cognitive flexibility abilities, while HCs showed the opposite pattern. The results suggest that emotion recognition abilities and cognitive flexibility performance are overall unrelated to moral reasoning level.

With respect to delinquency, moderate and positive correlations were found in KS patients in the ‘self’ condition and the ‘unpersonal other’ condition, suggesting that lower reasoning levels in these conditions are related to a higher tendency of engaging in delinquent behavior in KS patients.

## 4. Discussion

The aim of this study was to evaluate the differences in moral decision-making and moral reasoning between KS patients and healthy controls by assessing moral dilemmas varying in self–other perspective. KS patients judged moral dilemmas as less wrong than HC and were more intended to perform everyday dilemmas; referred to as a utilitarian bias. Moreover, moral reasoning levels were generally lower in KS patients than controls, especially when dilemmas related to their own behavior. In contrast, the HC group showed the highest moral maturity levels when motivating their own choices compared to moral decisions for others. Self-reported delinquent behavior premorbid to KS was higher in the KS group as compared to the HC group. Interestingly, moral reasoning levels in KS patients correlated negatively with premorbid delinquency, thereby demonstrating an important role for premorbid attitudes prior to the development of KS, regarding current (un)desirable moral behavior postmorbid.

Regarding the primary objective of the current study, we found that both moral decision-making and moral reasoning abilities are lower in KS patients. More specifically, KS patients tended to show a utilitarian bias, which is more in line with research executed in patients with frontal lobe damage resulting from traumatic brain injury (TBI) and other neurological and psychiatric patient groups [16,18,19,40]. In KS patients, this bias is illustrated by a higher tendency in KS patients to perform the dilemmas themselves compared to healthy controls. Moreover, KS patients judged dilemmas as “less wrong” when compared to HCs. A possible reason why we did find evidence for the utilitarian bias, in contrast to the earlier study [20], is that we specifically distinguished between moral judgements of own behavior and judgements of behaviors of (un)personal others. Regarding decision-making, this distinction resulted in finding a fluctuation in KS patients when making a judgement on wrongness depending on the familiarity of the actor. Namely, KS patients considered the behavior of a personal other least wrong, followed by their own behavior, and lastly, that of unpersonal others. In the ‘unpersonal other’ condition, difference in the evaluation of wrongness is not significant, leading to the impression that (moral) law obedience in the KS and HC groups are equal here and thus similarly present in KS patients as well. Therefore, this result might be considered as a clear sign of a utilitarian bias. A second possible reason for observing a utilitarian bias in KS patients is that we explicitly cued KS patients and healthy controls to think about the consequences of the action by asking how wrong it was to perform the action, while the earlier study did not incorporate this. As Dooley and colleagues [40] showed, young patients with frontal TBI also did not benefit from receiving additional social cues as KS patients in our study did. The finding that the behavior of a personal other is considered least wrong according to KS patients, might be explained by the relative lack of social connections KS patients experience, leading to a higher value of the relatives compared to themselves [7]. Regardless, the results indicate altered decision-making in KS patients when compared to HCs, whose judgements remain similar in the three conditions of the MBI.

We also found differences in moral maturity levels between the two groups across the three conditions of the MBI, which is consistent with the earlier study on this topic [20]. We found, in all three conditions, mean reasoning level to be lower in the KS group. The most striking result, however, is that the ‘self’ condition reasoning level in KS patients was the lowest, while HCs showed the highest maturity level. As with decision-making, the ‘unpersonal other’ condition showed the smallest group difference. These results provide further evidence for differences in judging moral situations between KS and HCs and points towards utilitarian judgements in KS patients when the actor is oneself or a proximal other. That is, adapting a more egocentric viewpoint by a higher willingness to judge moral violations as acceptable when it helps reaching goals [13,41].

As the field of forensic neuropsychology is gaining interest, we believe this study is a starting point for a line of research on moral development, adult criminal attitudes, and neurological disorders. We found a moderate to strong relationship between moral decision-making, moral reasoning, and premorbid delinquency. This result suggests consistency in moral attitudes throughout life in KS patients. A longitudinal study in adolescents and young adults also pointed to the consistency and reciprocity of moral reasoning levels in relation to delinquency and found consistent modest negative correlations between delinquency and moral reasoning [42]. This statement is in line with the results of the current study, implying that (im)moral attitudes present before the onset of the KS diagnosis, playing a role in current moral functioning. Immoral attitudes seem to be present premorbid instead, rather than being a consequence of the diagnosis. This raises the question if moral development plays an etiological role in the development of the disorder, or if other factors might co-account for the current finding. For example, developmental determinants of criminal behavior (e.g., family factors and peer influences), life-events, and attitudes on criminal behavior might be relevant [43,44]. To our current knowledge, this is the first study pointing towards early premorbid delinquent attitudes, moral maturity, and later neurological disease.

## 5. Limitations

Several limitations need to be considered when interpreting our results and making recommendations for follow-up research. First, a larger sample size would be desirable, as it would strengthen our finding and increase distribution of delinquency scores. In this study, delinquency scores were significantly higher in the KS sample, but not high in an absolute sense when looking at the maximum obtained score of the questionnaire. Second, in future research, it would be relevant to include not only self-reports of delinquency, but also more objective standards. Because of the confidential nature, we did not have access to criminal records of the controls and patients. True criminal offences are therefore uncertain and might also be omitted due to socially desirable answers or episodic memory deficits, having its effects on the results. However, because the relationships between morality and delinquency were quite strong, we have reasons to assume that social desirability was not fully explaining the relationship between delinquency and moral functioning. Regarding memory deficits, according to the Ribot curve [3,45] episodic memory function of early adulthood can be considered relatively good. Therefore, self-reported delinquent behaviors of this period of life can be considered sufficient. In contrast, delinquent behaviors of later in life are at greater risk to be forgotten, implying that true criminal offences might even be underestimated in the KS group. A final recommendation for future research would be to include an atypical KS group (i.e., non-alcoholic). However, as only very few cases are identified annually, it is difficult to compose a non-alcoholic KS group. In this particular population, mainly case studies are described. This led to the consideration of not including this population in the current study.

## 6. Conclusions

The current study demonstrates that KS patients show reduced moral decision-making and reasoning abilities, especially when situations relate to themselves. Furthermore, KS patients are more intended to perform moral questionable behavior, reflective of a utilitarian bias. Risk factors for immoral behaviors seem to originate mainly before the KS diagnosis, as we found a correlation with premorbid delinquent behavior. The results emphasize that KS patients are herewith at risk for unthoughtful socio-moral decisions and maladaptive behavior, possibly based on traits exhibited prior to the development of KS. The current study underlines the role of a healthy developmental environment in which moral development can take place in an optimal way. As illustrated, suboptimal moral maturity seems to be a risk factor for continuing immoral attitudes leading to undesirable behaviors.

## Figures and Tables

**Table 2:** Performance on neuropsychological tasks and the Delinquency Questionnaire in Korsakoff patients and healthy controls.

Task	KS (n = 20)	HC (n = 20)	Statistics
BADS RSC Test profile score (M, SD)	1.95 (1.47)	3.16 (0.83)	*U* = 100.50, *z* = −2.60, *p* < 0.001
D-KEFS CWIT error scaled scores (M, SD)	3.65 (3.56)	10.90 (2.34)	*U* = 21.50, *z* = −4.93, *p* < 0.001
ERT Total Residual Score (M, SD)	10.89 (6.00) (*n* = 19)	−4.94 (8.16) (*n* = 18)	*t*(35) = −2.45, *p* = 0.020
DQ total score (M, SD)	6.50 (5.32)	0.80 (1.67)	U = 47.50, *z =* −4.30, *p* < 0.001
DQ violent offences (M, SD)	2.95 (2.46)	0.45 (1.05)	*U* = 49.00, *z* = −4.30, *p* < 0.001
DQ non-violent offences (M, SD)	3.70 (3.03)	0.35 (0.99)	*U* = 58.00, *z* = −3.96, *p* < 0.001

Notes: RSC = BADS Rule Shift Cards Test; CWIT = D-KEFS Color Word Interference Test (fourth condition); ERT = Emotion Recognition Task; KS = Korsakoff’s syndrome; HC = healthy controls.

**Table 3:** Spearman’s correlations between emotion recognition, cognitive flexibility, and delinquency in different conditions of moral decision-making in Korsakoff’s syndrome patients and healthy controls.

Variable	Group (Sample Size)	Imaginary Performance	Self-Condition	Personal Other Condition	Unpersonal Other Condition
Emotion Recognition (*r_s_, p*-value)	KS (n = 19)	−0.25, *p* = 0.311	0.30, *p* = 0.212	−0.094, *p* = 0.702	−0.12, *p* = 0.634
	HC (n = 18)	14, *p* = 0.592	0.06, *p* = 0.813	−0.02, *p* = 0.925	−0.02, *p* = 0.951
Cognitive Flexibility (*r_s_*, *p*-value)	KS (n = 20)	−0.09, *p* = 0.714	0.12, *p* = 0.612	0.07, *p* = 0.783	0.32, *p* = 0.175
	HC (n = 20)	0.29, *p* = 0.210	0.09, *p* = 0.712	−0.19, *p* = 0.415	−0.29, *p* = 0.415
Delinquency (r_s_, *p*-value)	KS (n = 20)	0.64, *p* = 0.003	−0.47, *p* = 0.037	−0.17, *p* = 0.475	−0.22, *p* = 0.348
	HC (n = 20)	−0.05, *p* = 0.834	0.33, *p* = 0.161	0.06, *p* = 0.787	0.14, *p* = 0.570

Notes: KS = Korsakoff’s Syndrome; HC = healthy controls.

**Table 4:** Spearman’s correlations between emotion recognition, cognitive flexibility, and delinquency in different conditions of Moral Reasoning in Korsakoff’s syndrome patients and healthy controls.

Variable	Group (Sample Size)	Self-Condition	Personal Other Condition	Unpersonal Other Condition
Emotion Recognition (*r_s_, p*-value)	KS (n = 19)	−0.36, *p* = 0.129	0.02, *p* = 0.929	0.25, *p* = 0.302
	HC (n = 18)	−0.13, *p* = 0.619	−0.17, *p* = 0.511	−0.57, *p* = 0.013
Cognitive Flexibility (*r_s_*, *p*-value)	KS (n = 20)	0.03, *p* = 0.906	0.10, *p* = 0.681	0.49, *p* = 0.028
	HC (n = 20)	0.02, *p* = 0.920	−0.11, *p* = 0.648	−0.49, *p* = 0.028
Delinquency (r_s_, *p*-value)	KS (n = 20)	−0.46, *p* = 0.043	−0.41, *p* = 0.072	−0.47, *p* = 0.038
	HC (n = 20)	0.03, *p* = 0.887	−0.04, *p* = 0.883	−0.26, *p* = 0.275s

Notes: KS = Korsakoff’s syndrome; HC = healthy controls.

## Data Availability

The data that support the findings of this study are available on request from the corresponding author. The data are not publicly available due to privacy or ethical restrictions.
